# Phase II study of concurrent chemoradiotherapy with capecitabine and cisplatin in patients with locally advanced squamous cell carcinoma of the head and neck

**DOI:** 10.1038/sj.bjc.6602849

**Published:** 2005-10-25

**Authors:** J G Kim, S K Sohn, D H Kim, J H Baek, S B Jeon, Y S Chae, K B Lee, J S Park, J H Sohn, J C Kim, I K Park

**Affiliations:** 1 Department of Oncology/Hematology, Kyungpook National University, College of Medicine, Kyungpook National University Hospital, Daegu, Korea; 2 Department of Otorhinolaryngology, Kyungpook National University, College of Medicine, Kyungpook National University Hospital, Daegu, Korea; 3 Department of Radiation Oncology, Kyungpook National University, College of Medicine, Kyungpook National University Hospital, Daegu, Korea

**Keywords:** capecitabine, chemoradiotherapy, cisplatin, head and neck cancer

## Abstract

We aimed to evaluate the efficacy and safety of concurrent chemoradiotherapy with capecitabine and cisplatin in patients with locally advanced squamous cell carcinoma of the head and neck (SCCHN). In total, 37 patients with stage III or IV SCCHN were enrolled on the study. The chemotherapy consisted of two cycles of intravenous cisplatin of 80 mg m^−2^ on day 1 and oral capecitabine 825 mg m^−2^ twice daily from day 1 to day 14 at 3-week intervals. The radiotherapy (1.8–2.0 Gy 1 fraction day^−1^ to a total dose of 70–70.2 Gy) was delivered to the primary tumour site and neck. The primary tumour sites were as follows: oral cavity (*n*=6), oropharynx (*n*=11), hypopharynx (*n*=8), larynx (*n*=3), nasopharynx (*n*=6), and paranasal sinus (*n*=3). After the chemoradiotherapy, 29 complete responses (78.4%) and 6 partial responses (16.2%) were confirmed. Grade 3 or 4 neutropenia occurred only in two patients, plus grade 3 febrile neutropenia was observed only in one patient. At a median follow-up duration of 19.8 months, the estimated overall survival and progression-free survival rate at 2-year was 76.8 and 57.9%, respectively. Concurrent chemoradiotherapy with capecitabine and cisplatin was found to be well tolerated and effective in patients with locally advanced SCCHN.

The majority of patients with squamous cell carcinoma of the head and neck (SCCHN) present a locoregionally advanced disease, which is associated with a poor prognosis despite treatment with surgical resection or radiation, or both (Vokes *et al*, 1993). Since the effects of treatment on functional abilities, such as speech and eating, are additional factors to consider in patients with SCCHN, recent attempts to improve the major end points of treatment (local control, organ preservation, and overall survival) have focused on the use of radiotherapy with concurrent chemotherapy (Brizel *et al*, 1998; Wendt *et al*, 1998; Calais *et al*, 1999; Pignon *et al*, 2000). Generally, cisplatin combined with 5-fluorouracil (5-FU) have been considered as one of standard regimen for concurrent chemoradiotherapy (Browman *et al*, 1994; Brizel *et al*, 1998; Wendt *et al*, 1998; Calais *et al*, 1999). However, the adverse effects of 5-FU, such as oral mucositis, which is an additive complication to radiation, or bone marrow suppression, can result in treatment-related hospitalisation or mortality, thereby compromising the quality of life and compliance to treatment (Wendt *et al*, 1998).

The oral fluoropyrimidine capecitabine (Xeloda®; Hoffmann-La Roche) was rationally designed to preferentially generate 5-FU in tumour tissue and mimic continuous-infusion 5-FU. This tumour selectivity is achieved through exploiting the significantly higher activity of thymidine phosphorylase (TP) in many tumour tissues compared with healthy tissue (Miwa *et al*, 1998; Schuller *et al*, 2000). The expression of this enzyme is enhanced in tumour areas with poor perfusion, hypoxia, and acidosis, a situation found in most advanced HNSCC. Moreover, there is evidence that radiation leads to the upregulation of TP expression (Sawada *et al*, 1999). In a preclinical study, capecitabine given orally resulted in consistently higher tissue-to-plasma 5-FU concentration ratios than 5-FU administered intravenously (Ishikawa *et al*, 1998). In addition, capecitabine has also exhibited antitumour activity when given as a monotherapy or in combination with cisplatin in patients with various solid tumours as well as in advanced HNSCC (Blum *et al*, 1999; Van Cutsem *et al*, 2001; Kim *et al*, 2002; Pivot *et al*, 2003; Park *et al*, 2004).

Furthermore, since the key side effects of capecitabine are hand–foot syndrome and diarrhoea, which overlap little with the side effects of cisplatin or radiation, capecitabine can be a good chemotherapeutic agent in concurrent chemoradiotherapy for SCCHN.

Accordingly, the current phase II study was conducted to evaluate the efficacy and safety of concurrent chemoradiotherapy with capecitabine and cisplatin for locally advanced SCCHN.

## PATIENTS AND METHODS

### Eligibility

All the patients involved in the current study had measurable, histologically or cytologically confirmed, locoregionally advanced stage III or IV SCCHN arising from the oral cavity, pharynx, larynx, or paranasal sinuses. The patients were 20–75 years of age with a performance status of 0–2 on the Eastern Cooperative Oncology Group (ECOG) scale. Plus, adequate haematological (WBC count ⩾4 × 10^9^ l^−1^, platelet count ⩾100 × 10^9^ l^−1^, haemoglobin ⩾9 g dl^−1^), renal (serum creatinine ⩽1.5 mg dl^−1^ and creatinine clearance ⩾50 ml min^−1^), and hepatic (total bilirubin ⩽2.0 mg dl^−1^ and serum transaminase level ⩽3 times the upper limit of the normal range) levels were also required. Patients were ineligible if they had previously received chemotherapy or radiation therapy, or had other severe medical illnesses, distant metastasis, another active malignancy in the last 5 years, except treated nonmelanoma skin cancer or cervical dysplasia, or a history of anaphylaxis to drugs. The institutional review board of the authors' institution approved the protocol, and written informed consent was obtained from all patients before enrollment.

### Study treatment

**Figure 1:**
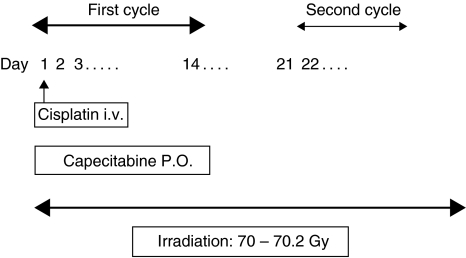
Administration schedule of concurrent chemoradiotherapy with capecitabine and cisplatin in patients with squamous cell carcinoma of the head and neck. i.v: intravenous; PO: per oral.

The administration schedule is shown in Figure 1. Capecitabine 825 mg m^−2^ b.i.d with pyridoxine 100 mg t.i.d was given on days 1–14, followed by a 7-day rest period. The capecitabine was supplied as film-coated tablets at two dose strengths, 150 and 500 mg, while the cisplatin 80 mg m^−2^ was administered through a 1-h intravenous infusion on the first day of each cycle. Pre- and post intravenous hydration and appropriate antiemetics were also administered to prevent renal toxicity and emesis. Two cycles of chemotherapy were repeated every 3 weeks.

Radiotherapy (1.8–2.0 Gy 1 fraction day^−1^ to a total dose of 70–70.2 Gy), administered 5 days per week, was delivered to the primary tumour site and neck, and was targeted to begin on the first day of chemotherapy. Patients underwent a complete dental evaluation and treatment as early as possible before the initiation of radiotherapy. Every effort was made to continue the radiation on schedule.

### Dose modification

The protocol plan was to continue the study treatment despite mucositis or dermatitis. However, if grade 3 or 4 capecitabine-related haematological or nonhaematological toxicity, such as diarrhoea and hand–foot syndrome, occurred (not including radiation-related toxicity), capecitabine was withheld until the toxicity had improved by at least two grade levels. Subsequent capecitabine doses then required a 20% dose reduction. The dose of cisplatin was reduced to 50% if the calculated creatinine clearance level was 30–50 ml min^−1^. No cisplatin was administered if the creatinine clearance level was less than 30 ml min^−1^. In the presence of myelosuppression (WBC count< 4 × 10^9^ l^−1^ or platelet count <100 × 10^9^ l^−1^), a persisting fever that exceeded 38°C, or other clinically apparent infections, a cycle could be postponed for 1 week or interrupted if this was judged to be necessary in the opinion of the attending physicians.

### Surgery

Salvage surgery of the primary tumour site was recommended for operable patients with a respectable disease, who failed to achieve a complete response (CR) after the end of the chemoradiotherapy. The extent of surgery varied, ranging from laser resection or a wide excisional biopsy to a complete resection of the oral cavity or tonsillar primary. A modified neck dissection could also be performed. The surgery was carried out routinely 6–8 weeks after the chemoradiotherapy.

### Study assessments

Before being enrolled on the trial, all patients underwent a full medical history and physical examination, blood tests, computed tomography (CT) or magnetic resonance imaging of the head and neck, and chest X-ray (CT of the chest if the patient's low neck nodes were involved). Assessment of the tumour response by clinical examination and CT scanning took place 6 weeks after completing the chemoradiation therapy. A biopsy or fine needle aspiration cytology to determine the pathologic response was not routinely performed. The definitions of CR, partial response (PR), stable disease (SD), and progressive disease (PD) were based on the standard definitions established by the WHO (1979). The patients were monitored for toxicity (medical interview, physical examination, and complete blood count) throughout the treatment. Complete blood counts and chemistry were performed every week until the end of the chemoradiotherapy. Systemic toxicity resulting from treatment was graded according to the National Cancer Institute Common Toxicity Criteria (NCI-CTC) version 3.0. Acute radiation toxicities were graded according to the European Organization Therapy Oncology Group (EORTC-RTOG) toxicity criteria. Hand–foot syndrome was graded 1–3, as defined in previous capecitabine clinical studies (Blum *et al*, 1999).

### Statistical analysis

As a phase II study, the primary end point was to evaluate the response rate, while toxicity, progression, and overall survival were the secondary end points. For sample size calculation, the current trial used a two-stage optimal design, as proposed by Simon (1989), with a 90% power to accept the hypothesis and 5% significance to reject the hypothesis. Plus, the current trial was designed to detect a response rate of 90% as compared to a minimal, clinically meaningful response rate of 70%. Allowing for a follow-up loss rate of 10%, the total sample size was 36 patients with a measurable disease. Time to progression was measured as the time from the initiation of therapy until death of disease or toxicity, appearance of new lesions, or a greater than 25% increase of the indicator lesions over the previous smallest size. Overall survival was measured from the initiation of therapy to the date of the last follow-up or any cause of death. Progression-free and overall survival analyses were all estimated using the Kaplan–Meier method. The statistical data were obtained using an SPSS software package (SPSS 11.0 Inc. Chicago, IL, USA).

## RESULTS

### Patient characteristics

A total of 37 patients were enrolled in the current study from April 2003 to May 2004 at Kyungpook National University Hospital, Daegu, Korea. The characteristics of the patients are summarised in Table 1. The median age of the patients was 61.0 years (range, 35–75 years), and 31 (83.8%) patients were male. Most of the patients (89.2%) had a good performance status (ECOG 1). The primary sites of the tumours were as follows: oral cavity (*n*=6), oropharynx (*n*=11), hypopharynx (*n*=8), larynx (*n*=3), nasopharynx (*n*=6), and paranasal sinus (*n*=3). A total of 22 patients (59.5%) had a stage III disease, while the remaining 15 patients were stage IV. In all, 16 patients (43.2%) had an N2 (*n*=15) or N3 (*n*=1) status before treatment.

### Response and survival

**Figure 2:**
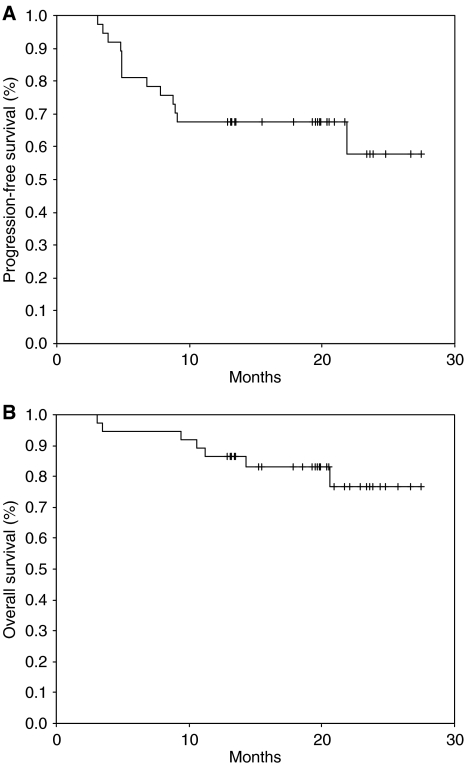
Progression-free survival (**A**) and overall survival (**B**) for all patients.

Of the 37 patients, 34 (91.2%) completed the planned treatment, with the remaining three being lost to follow-up or patient refusal. All efficacy data are reported using the intent-to-treat patient population. After the chemoradiotherapy, 29 CRs (78.4%) and 6 PRs (16.2%) were confirmed, giving an overall clinical response rate of 94.6% (95% CI; 87.0–102.2%). The primary site CR was 80.0% (28 out of 35) and metastatic lymph node CR was 70.4% (19 out of 27) (Table 2). Four out of six patients with nasopharyngeal tumour showed CR and remaining two patients PR. Among the eight patients who failed to achieve CR after the chemoradiotherapy, three patients underwent surgery and two patients received salvage chemotherapy. At the time of the present evaluation, 13 patients had developed disease progression or recurrence (five – primary tumour, three – regional lymph node, two – both primary tumour and regional lymph node, and three – distant metastases to the bone), and seven patients had died of disease progression. The median survival time had not yet been reached at a median follow-up duration of 19.8 months (range, 3.1–27.5 months), while the estimated overall survival and progression-free survival rate at 2-year was 76.8±8.5 and 57.9±11.1%, respectively (Figure 2). Locoregional control rate of the disease at 2-year was 72.6±7.4%. For the patient group except those with nasopharyngeal tumor, the estimated overall survival and progression-free survival rate at 2-year was 81.3±9.8 and 74.2±7.9%, respectively.

### Toxicity

All 37 patients were assessable for toxicity. The haematologic and nonhaematologic toxicities that occurred during the current study are summarised in Table 3. The most severe haematologic adverse event was neutropenia, which occurred with a grade 3/4 intensity in two patients (5.4%). Plus, grade 3 febrile neutropenia was observed in one patient (2.7%). Although this case was successfully treated with antibiotics and G-CSF, the patient withdrew their consent after this experience. No treatment-related death occurred during this study. Mucositis and dermatitis, as expected from a combination of radiation with an effective chemotherapeutic-sensitising agent, were the most common nonhaematological toxicities. Grade 3/4 mucositis and dermatitis was observed in 67.6 and 24.3%, respectively. Grade 2 hand–foot syndrome, a complication of capecitabine, occurred only in four patients (10.8%). The dose of capecitabine was reduced in two cycles due to neutropenia or diarrhoea, and cisplatin omitted from one cycle because of nephrotoxicity. The second cycle of chemotherapy was delayed in nine patients for the following reasons: haematological toxicity (*n*=7), persistent fever (*n*=1), and patient refusal (*n*=1). The dose intensity of capecitabine and cisplatin was well maintained throughout the study cycles.

## DISCUSSION

Many studies have demonstrated chemotherapy and radiotherapy to be highly effective in increasing the survival of patients with an unresectable disease. Moreover, concurrent chemoradiotherapy and induction chemotherapy have been established as an appropriate standard of care for patients with locally advanced SCCHN. However, no standard concurrent chemoradiotherapy regimen has been defined.

Therefore, the present phase II study was designed to evaluate the efficacy and toxicity of capecitabine instead of 5-FU, a commonly used agent, in combination with cisplatin for concurrent chemoradiotherapy in patients with locally advanced SCCHN. In the current study, the clinical CR rate (78.4%), locoregional control rate (72.6% at 2-year), and progression-free survival rate (57.9% at 2 years) following treatment with the present regimen, which can be administered on an outpatient basis, were comparable with previous results reported for 5-FU and platinum-based concurrent chemoradiotherapy, although the follow-up period was relatively short to compare the survival rate directly (Calais *et al*, 1999; Adelstein *et al*, 2000, 2003). For example, concurrent chemotherapy with infusion of 5-FU and cisplatin arm achieved a CR rate of 49.4% and 3-year overall survival rate of 27% in a randomised study compared with concurrent chemoradiotherapy with radiation therapy alone (Adelstein *et al*, 2003).

Since the efficacy and favourable safety profile of capecitabine have been clearly demonstrated in recent large phase III studies comparing capecitabine with intravenous 5-FU plus leucovorin for metastatic colorectal cancer (Hoff *et al*, 2001; Van Cutsem *et al*, 2001), capecitabine has been widely used in the treatment of breast cancer, stomach cancer, and other solid tumours (Blum *et al*, 1999; Kim *et al*, 2002, 2003). Capecitabine also offers a number of potential advantages as a chemoradiosensitiser in concurrent chemoradiotherapy. Daily administration mimicking the continuous infusion of 5-FU can act as a radiosensitiser for every fraction of radiotherapy. Furthermore, its mode of activation by TP and radiotherapy concentrates it within tumour cells, raising the prospect of better tumour control. Given these advantages, several studies have demonstrated that concurrent chemoradiotherapy using capecitabine, with a dose ranging from 800 to 825 mg m^−2^ b.i.d, in combination with cisplatin or oxaliplatin is effective and has a low toxicity profile in the neoadjuvant setting of rectal cancer or locally advanced oesophageal cancer (Rodel *et al*, 2003; Hofheinz *et al*, 2005; Kim JC *et al*, 2005; Kim SB *et al*, 2005).

Oral mucositis and myelosuppression are the most serious complications of concurrent chemoradiotherapy for SCCHN, resulting in a reduced compliance to treatment or sometimes mortality. In the present study, mucositis was the most common adverse effect observed, with a grade 3 or 4 intensity in 67.6% of the patients. In a randomised study by Calais *et al* (1999), the incidence of grade 3 or 4 mucositis in concurrent chemoradiotherapy with 5-FU and cisplatin was 71%, which was higher than with radiotherapy only (39%). The incidence of mucositis was not so different between chemoradiotherapy with capecitabine/cisplatin and 5-FU/cisplatin. Meanwhile, grade 3 or 4 neutropenia occurred only in two patients (5.4%), plus grade 3 febrile neutropenia was observed in patient (2.7%) in the current study. These incidences of haematologic toxicities were significantly different from previous studies using 5-FU-containing regimens, where the incidence of grade 3 or 4 leukopenia was 29–81% (Vokes *et al*, 2000; Adelstein *et al*, 2003). Recently, taxanes, such as paclitaxel and docetaxel, which exhibit activity against SCCHN, are being increasingly used for concurrent chemoradiotherapy to improve the treatment outcome (Kies *et al*, 2001; Tishler *et al*, 2002; Katori *et al*, 2004). Kies *et al* (2001) reported that concomitant infusional paclitaxel and fluorouracil, oral hydroxyurea, and radiation therapy achieved a clinical CR rate of 69%, 3-year locoregional control rate of 86%, and 3-year overall survival rate of 60% in patients with locally advanced SCCHN. Thus, when considering the high percentage of patients with a T4 and N2/3 disease in this study, the locoregional control rate and overall survival seemed to be promising. However, 34% of the patients experienced grade 3 or 4 leukopenia, and two patients (3%) died of treatment-related toxicities. Therefore, taxane-based regimens should be tested in a randomised trial and compared with a less intensive concurrent regimen.

In conclusion, concurrent chemoradiotherapy with capecitabine and cisplatin was found to be well tolerated and effective in patients with locally advanced SCCHN. Accordingly, this regimen can be regarded as an important chemoradiotherapy option for advanced HNSCC, although long-term follow-up is needed to evaluate the late treatment failure and complications.

## Figures and Tables

**Table 1:** Patient characteristics

**Characteristic**	**Number of patients, *N*=37 (%)**
*Age (years)*	
Median (range)	61 (35–75)
	
Male/female	31 (83.8)/6 (16.2)
	
*ECOG performance status*	
1	33 (89.2)
2	4 (10.8)
	
*Site of primary tumour*	
Oral cavity	6 (16.2)
Oropharynx	11 (29.7)
Hypopharynx	8 (21.6)
Larynx	3 (8.1)
Paranasal sinus	3 (8.1)
Nasopharynx	6 (16.2)
	
*Histologic classification*	
Well differentiated	8 (21.6)
Moderately differentiated	10 (27.0)
Poorly or undifferentiated	11 (29.7)
Unspecified	8 (21.6)
	
*Stage*	
III	22 (59.5)
IV	15 (40.5)
	
*T classification*	
T1	3 (8.1)
T2	15 (40.5)
T3	13 (35.1)
T4	6 (16.2)
	
*N classification*	
N0	9 (24.3)
N1	12 (32.4)
N2	15 (40.5)
N3	1 (2.7)

**Table 2:** Tumour response (intent-to-treat analysis, *N*=37)

	**Response (%)**				
**Variable**	**CR**	**PR**	**SD**	**PD**	**Response rate**
Primary site	28/35 (80.0)	5/35 (14.3)	2/35 (5.7)	0/35	33/35 (94.3)
Lymph nodes	19/27 (70.4)	5/27 (18.5)	2/27 (7.4)	1/27 (3.7)	24/27 (88.9)
Overall	29/37 (78.4)	6/37 (16.2)	1/37 (2.7)	1/37 (2.7)	35/37 (94.6)

CR=complete response; PR=partial response; SD=stable disease; PD=progressive disease.

**Table 3:** Acute toxic effects (*N*=37)

	**Grade (% of patients)**				
	**1**	**2**	**3**	**4**	**Grade 3/4 (%)**
*Haematologic*					
Anaemia	13 (35.1)	6 (16.2)	2 (5.4)	1 (2.7)	8.1
Leukopenia	6 (16.2)	8 (21.6)	2 (5.4)	1 (2.7)	8.1
Neutropenia	7 (18.9)	6 (16.2)	1 (2.7)	1 (2.7)	5.4
Thrombocytopenia	12 (32.4)	2 (5.4)	1 (2.7)		2.7
Febrile neutropenia			1 (2.7)		2.7

*Nonhaematologic*					
Nausea	11 (29.7)	12 (32.4)	5 (13.5)	1 (2.7)	16.2
Vomiting	9 (24.3)	10 (27.0)	3 (8.1)		8.1
Mucositis	2 (5.4)	10 (27.0)	17 (45.9)	8 (21.6)	67.6
Dermatitis (in-field)	3 (8.1)	12 (32.4)	7 (18.9)	2 (5.4)	24.3
Diarrhoea	4 (10.8)	3 (8.1)	1 (2.7)		
Hand–foot syndrome	12 (32.4)	4 (10.8)			
